# Using a behavioural framework to optimize antibiotic prescribing by family medicine residents

**DOI:** 10.15694/mep.2021.000113.1

**Published:** 2021-05-09

**Authors:** Samantha Moe, Tiffany Kan, Charlene Soobiah, Azy Golian, Timothy Li, Sumit Raybardhan

**Affiliations:** 1 The College of Family Physicians of Canada; 2 North York General Hospital; 3 University of Toronto; 4 North York General Hospital

**Keywords:** antibiotic prescribing, medical trainee, behaviour change wheel, theoretical domains framework, family medicine

## Abstract

This article was migrated. The article was marked as recommended.

**Background and objectives:**Overprescribing of antibiotics in primary care is a prominent concern in the context of increasing antimicrobial resistance worldwide. Medical trainees are a key group to deliver thoughtful antimicrobial stewardship training. This study examined the factors influencing antibiotic prescribing for upper respiratory tract infections (URTI) by family medicine residents in order to identify educational interventions.

**Methods:** Using purposive sampling of family medicine residents, semi-structured interviews were conducted until thematic saturation was reached. Interviews were coded into the domains of the Theoretical Domains Framework (TDF). Belief statements were created to characterize each domain and categorized as enablers or barriers to appropriate prescribing. Domains were plotted on the Behaviour Change Wheel (BCW) and intervention functions identified.

**Results:**Twelve participants were interviewed. Nine domains of the TDF were relevant to antibiotic prescribing. Social influence was a prominent theme with the preceptor and patient being key influences on resident prescribing. Learning goals were also a key theme including the desire to strengthen independent clinical decision-making skills and improve antibiotic knowledge. Residents’ beliefs about capabilities were challenged when faced with diagnostic uncertainty. Additional domains included: professional role; environmental context and resources; intentions; beliefs about consequences and capabilities, and knowledge. Using the BCW, nine intervention functions were identified to change antibiotic prescribing behaviour.

**Conclusion:** This study found nine domains of the TDF were relevant to family medicine resident antibiotic prescribing for URTI. Nine intervention functions could be used to guide intervention design.

## Introduction

Antimicrobial resistance is a public health priority worldwide, as many bacteria previously susceptible to antibiotics have evolved into resistant pathogens ( World Health Organization, 2014). Attention has recently turned to antibiotic use in primary care, as the majority of antibiotics are prescribed in this setting (Canadian Antimicrobial Resistance Alliance, 2015). A recent US study estimated that 30% of outpatient antibiotic prescriptions may have been inappropriate ( Fleming-Dutra *et al*., 2016).

Numerous studies have sought to identify what barriers lead to deleterious antimicrobial prescribing behaviours. A systematic review identified several factors that impact antibiotic misuse including physicians’ fear of serious patient complications; diagnostic uncertainty; or time pressures ( Teixeira Rodrigues *et al.*, 2013). Dozens of studies have attempted to address these barriers using a variety of interventions, such as audit and feedback, and patient or provider education ( Hrisos *et al.*, 2008; Butler *et al.*, 2012; Gerber *et al.*, 2013). These studies have shown modest, if any, benefit. Many of the studies used an “intuitive” intervention design, in that the attitudes, behaviours and beliefs that underpin antibiotic prescribing were not considered, potentially contributing to the modest effects observed. In a systematic review conducted by Charani and colleagues, of 191 studies that aimed to improve antimicrobial prescribing, only 10 primary studies addressed behaviour change ( Charani *et al.*, 2011). A report published by Public Health England called for interventions grounded in behaviour change therapy to improve upon outcomes in this area ( Public Health England, 2015).

Antimicrobial stewardship training has been incorporated widely into medical education at the undergraduate and postgraduate level ( Silverberg *et al.*, 2017). Arguably, interventions designed for medical learners should also be grounded in an understanding of their attitudes, beliefs and behaviours in order to achieve sustainable results. While some papers have identified the beliefs and attitudes held by medical residents about antibiotic prescribing, to our knowledge, none have used the Behaviour Change Wheel (BCW), which is a validated framework that assesses behaviours and identifies interventions to change behaviour.

The purpose of this study was to examine the factors influencing family medicine residents’ antibiotic prescribing behaviour for upper respiratory tract infections (URTI) using the Theoretical Domains Framework (TDF). The Behaviour Change Wheel (BCW) was used to identify interventions to improve antibiotic prescribing. The Affordability, Practicability, Effectiveness, and Cost Effectiveness, Acceptability, Side Effects and Equity (APEASE) criteria ( Michie, van Stralen and West, 2014) was applied to identify optimal interventions at our site.

## Methods

A qualitative study was performed using an iterative and thematic approach to code and analyze data. The reporting of this study follows the recommendations from the COREQ (Consolidated criteria for reporting qualitative research) statement ( Tong, Sainsbury and Craig, 2007). The research team included three pharmacists from the Family Medicine and Antibiotic Stewardship programs at North York General Hospital; two family medicine residents from the family medicine residency program; and a research coordinator.

### Sampling and Participants

Purposive sampling of family medicine residents was done based on year of training, gender and International Medical Graduate (IMG) status. IMG physicians received their medical degree outside of Canada and are seeking licensure by completing residency in Canada. Inclusion of IMG physicians was to ensure their perspective was included. All residents were in their first or second year of postgraduate medical training at North York General Hospital, Toronto, Ontario. Recruitment of residents occurred via email and in person. Interviews were conducted by the research coordinator [CS], who has a qualitative research background and no previous involvement with participants. Written informed consent was obtained prior to interview. Interviews were conducted by phone, audio recorded and transcribed verbatim by the research coordinator. Participants were compensated with a $10 gift card.

### Data Collection

A semi-structured interview guide was drafted by one researcher [TK] and reviewed by two others [SM, SR] (see Supplementary File 1). The questions were based on the domains of the TDF( Cane, O’Connor and Michie, 2012) and adapted from the questionnaire developed by Dallas *et al.* ( Dallas *et al.*, 2014). The first section included questions about factors that affect prescribing antibiotics for URTI. The second section included questions about residents’ preferences for educational resources. Interview questions were piloted on two medical trainees [AG, TL] and modified for clarity and focus. No pilot interviews were used in the final data set. Transcripts were anonymized, transcribed, and proofread for accuracy [CM, SM].

Ethical approval was obtained from North York General Hospital (REB#16-0041) prior to recruitment. This study was funded by the Exploration Fund Grant, North York General Hospital, Toronto, ON, Canada.

### Analysis

The Theoretical Domains Framework (TDF), developed by Cane *et al.* ( Cane, O’Connor and Michie, 2012), is an integrated framework that distills behaviour change theories into a unified framework. The TDF is a validated method for assessing health related behaviours as a basis for intervention development. The BCW is a model that links the domains of the TDF to intervention functions ( Michie, van Stralen and West, 2011).

As interviews were conducted, preliminary analysis of the data began, and the interview guide was iteratively refined to explore emerging themes in more detail.

Data were coded into one or more domains of the TDF using NVivo, Version 11. All researchers coded two interviews together to develop a coding strategy. Belief statements were created to characterize each domain and were agreed upon by the research team. Each belief statement captured a core thought identified by interview participants ( Patey *et al.*, 2012) and each domain could have several belief statements. The remaining interviews were coded by researchers using the belief statements for guidance. Disagreements about coding were resolved through discussion.

**Figure 1.**
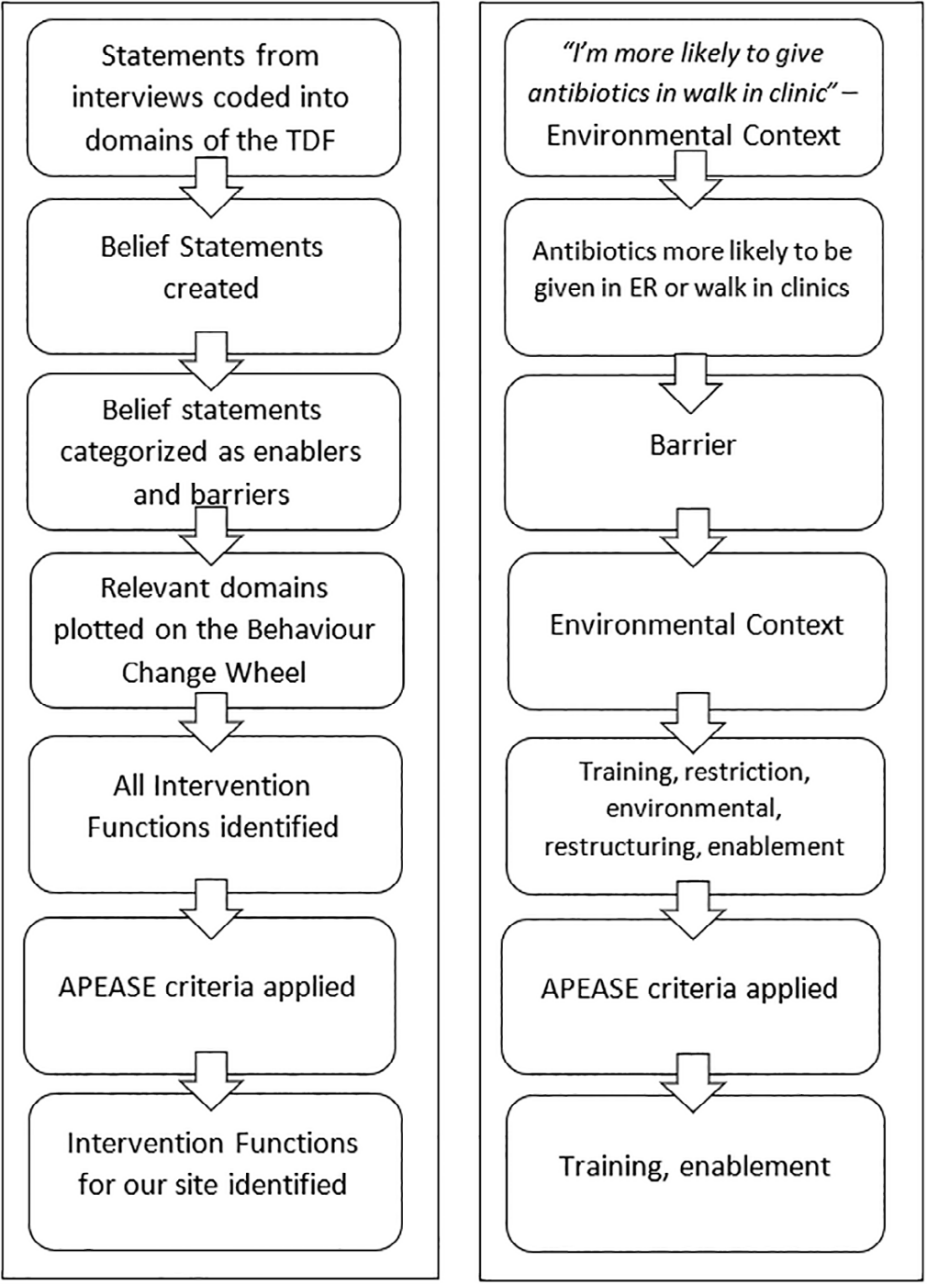
Description of Methods.

Interviews were conducted until thematic saturation was reached. A final two interviews were conducted to ensure that no new themes emerged. Belief statements were further refined once final interviews were analyzed. These statements were categorized as either enablers or barriers to appropriate antibiotic prescribing for URTI ( French *et al.*, 2012). Domains were plotted on the BCW ( Michie, van Stralen and West, 2011) and intervention functions were identified. The APEASE criteria were applied to identify the intervention strategies applicable to our practice site ( Michie, 2014). See Figure 1 for an illustration of methods and an example. The left column describes the process used to identify intervention functions.

## Results/Analysis

### Characteristics of Participants

A total of 12 participants (6 female; 6 male) from post-graduate year 1 (n=7) and year 2 (n=5) participated in the semi-structured interviews. Three residents were IMG residents with years in practice ranging from 0 to 5 years. Interviews took an average of 24 minutes.

### Key Themes Identified within the Domains of the TDF

Several themes were identified and mapped to the domains of the TDF (see Supplementary File 2). Overall, nine domains of the TDF were found to be relevant to antibiotic prescribing practices of family medicine residents based on frequency of statements, impact, and generalizability across residents. The domains included: clinical decision-making; environmental context and resources; intentions; beliefs about capabilities; professional role; social influences; beliefs about consequences, goals; and knowledge. The remaining five domains were less relevant, including emotions, behaviour regulation, reinforcement, skills and optimism.

### Clinical decision-making

Clinical decision-making included statements about using skills and knowledge to diagnose if an infection is bacterial or viral, and to decide if antibiotic should be prescribed.

Generally, residents avoid prescribing antibiotics for viral URTI. However, they are more likely to prescribe antibiotics for URTI when patients have risk factors including smoking, immunosuppression, or diabetes. Other factors that influenced prescribing included extremes of age, severity of presenting illness, or longer duration of illness.

### Environmental context and resources

Environmental context and resources included factors in the workplace that influenced residents’ prescribing practices and were mentioned by several residents. Residents indicated that access to diagnostic imaging and lab work influence their clinical decision-making. Other factors included the availability of antibiotics from local pharmacies; the patient’s ability to pay for prescriptions; and local resistance patterns. Antibiotic prescriptions were also more likely to be given if lack of follow-up was anticipated.

### Intentions

Intentions were reflections on how participants would act when confronted with either a viral or bacterial infection and were mentioned by all participants. In many cases, if a viral infection was suspected, participants stated they would not give an antibiotic and would follow up closely with the patient. If there was evidence of a bacterial infection, participants would prescribe an antibiotic. A few participants mentioned that they would not prescribe antibiotics for viral infections because they do not want to increase bacterial resistance.

### Beliefs about capabilities

Beliefs about capabilities were residents’ thoughts about their abilities to perform as a physician. Most residents feel confident differentiating between bacterial and viral infections. Generally, residents also feel comfortable when deciding whether to prescribe antibiotics for URTI, and which antibiotic to prescribe. Participants felt that their confidence increases during the course of their medical training. However, residents feel less confident about whether to prescribe antibiotics when patient follow-up is not available; when it is unclear if the etiology of infection is viral or bacterial; or when results of investigations conflict with the clinical picture.

Despite generally feeling comfortable with antibiotic prescribing, residents identified areas of antibiotic knowledge that could be improved upon, such as selection of antibiotic in specific scenarios including pregnancy, allergy, or treatment failure.

### Professional role

The domain of professional role related to the identity of being a family physician. Two main themes emerged under professional role. First, participants’ education as a result of being part of a professional program was coded under this domain. Resident’s learning about the diagnosis and management of URTI is significantly influenced by the education received during medical school and residency. In these settings, they learn about principles of treatment, clinical approach and practice customs.

The second theme related to the sense of responsibility residents feel about ensuring antibiotics were appropriately prescribed. Many residents feel responsible for ensuring antibiotics are used appropriately and educating patients about the same.

### Social influence

Social influence were interactions with individuals that affected the way residents think or practice. The most prominent theme under social influence was the relationship with the preceptor. Participants learned common practices by observing preceptor behaviour or discussing prescribing choices. In some circumstances, preceptors modelled antibiotic prescribing that residents found to be inconsistent with current recommendations. Residents also describe situations in which their preceptor’s prescribing practice was more liberal than expected, or discordant with previous teaching. When the preceptor’s prescribing practices were more liberal, residents found it challenging to avoid prescribing antibiotics for their preceptor’s patients.

Patient preference was also a prominent theme under the social influence domain. Patient preferences were often cited as a factor when prescribing antibiotics. Some residents described scenarios in which antibiotic prescriptions were provided to insistent patients presenting with a viral illness. Other residents described strategies such as re-education or making compromises to avoid prescribing antibiotics to patients with viral illnesses.

### Beliefs about consequences

Statements related to expectation of outcomes, consequences or regrets were captured under the domain of beliefs about consequences. Residents believed that if they stopped prescribing antibiotics for viral infections that most patients would get better on their own. Residents also believed this practice would lower drug expenditures and the risk of developing resistant pathogens. However, residents felt that patients with comorbidities or risk factors have the potential to get sick very quickly from URTIs and may experience poor outcomes if antibiotics were avoided completely.

### Knowledge of resources

The domain of knowledge was defined as an explicitly stated awareness of something. Most residents were aware of a variety of guidelines and medical databases to support their learning about infectious diseases and antibiotics. Residents find that antibiotic treatment guidelines tailored to local resistance patterns are particularly useful to guide prescribing.

### Goals

Goals were personal outcomes that residents wanted to achieve. Many residents felt confident about their clinical decision making ability. However, some felt that ongoing development of these skills was needed including their knowledge about antibiotic selection and how local antibiotic resistance impacts prescribing. Residents also identified the importance of developing independent decision-making skills during residency.

### Identifying Interventions

Once the nine domains were plotted on the BCW, nine intervention functions were identified as possibilities to change antibiotic prescribing behaviour. These included restriction, environmental restructuring, modelling, enablement, education, persuasion, incentivization, coercion and training ( Table 1). Since all nine intervention functions were deemed possible, the APEASE criteria was used to guide intervention selection ( Michie, 2014). The interventions of education and training, modelling, and enablement could address multiple domains and were feasible on-site.

**Table 1.** Summary of domains, enablers, barriers and intervention functions for antibiotic prescribing for family medicine residents.

Domains	Selected Interview Excerpts	Selected Enablers and Barriers that could be targeted for Interventions	Intervention Functions
**Social Influence**	*“The preceptors I am around are much more likely to treat with antibiotics than I am, ...so that’s kind of the feedback I have received from them ... I feel that I am prescribing more because of that, even when I don’t definitely know if someone has something bacterial.” (FMR 7)*	Residents learned about the management of URTI from preceptors. Occasionally, residents observed preceptor prescribing practices to be incongruent with current guidelines.When a preceptor’s antibiotic prescribing practice was liberal, residents found it challenging to avoid prescribing antibiotics.	Restriction Environmental Restructuring Modelling Enablement
**Beliefs about Capabilities**	*“When somebody looks ... unwell or has signs on an exam but their x-ray is negative, I feel a little less confident.” (FMR 5) “[S]he was ... a demanding kind of patient.... Then later when the x-ray came back a couple of days later it was normal, but we treated her anyway because she really wanted to be treated.”* *(FMR 5)*	Residents feel less confident about the decision to prescribe antibiotics when there is no follow up available, diagnosis is ambiguous, or etiology of the infection unclear. Residents find it challenging to manage patient expectations if different from their own desire to not give antibiotics.	Education Persuasion Modelling Enablement
**Goals (Knowledge and Clinical Decision Making)**	*“I guess there is like a typical first line ones that you feel comfortable prescribing but ... when people say they are allergic to this and what would be the next line. ...* *[S]ometimes I have to think twice about what you would give in pregnancy ...[A]lmost always [I] will look things up just to 100% confirm, so I would say my confidence level would be somewhat.” (FMR 4)* *“I guess [the decision to prescribe antibiotics or not was] more difficult earlier on but it’s become easier as time... like now that I’m a PGY2.” (FMR 6)*	Residents find treatment guidelines that include local prescribing considerations helpful. Residents identify areas of learning that can be improved upon: when to prescribe antibiotics; antibiotic selection; and understanding resistance data. Residents identify process of developing independent clinical decision-making skills. This is developed by considering their beliefs, values, medical literature/teaching and observed practices during training.	Education Training Environmental restructuring Enablement
**Professional Role**	*“It’s our job to educate people about the resistance rates, about side effects of antibiotics, and when they will not help. Then people understand, and we can reduce the resistance issue across the globe.” (FMR 6)*	Residents feel it is their professional responsibility to ensure antibiotic prescribing is appropriate and to educate patients.	Education Persuasion Modelling
**Intentions**	*“...the ones that are borderline [cases] ..., I would talk about resistance patterns and our reasons why we choose not to prescribe an antibiotic...” (FMR 4)*	Residents do not intend to prescribe antibiotics for viral infection. Residents want to prevent unnecessary side effects and avoid increasing antimicrobial resistance.	Education Persuasion Incentivization Coercion Modelling
**Beliefs about Consequences**	*“If we treat everybody with an antibiotic for a viral infection or interventions to prevent [superinfections] then we’d be doing more harm than good.” (FMR 3)*	Generally, residents believe that if they stop prescribing antibiotics for viral infections, there would be few negative consequences. However, they are more likely to prescribe antibiotics for URTI when comorbidities, risk factors or extremes of age are present out of concern of negative health outcomes. (Intentions - secondary consideration).	Education Persuasion Modelling
**Environmental Context**	*“...if I am at a walk-in clinic and I am not going to have follow up with the patient...” (FMR 7)*	Antibiotic prescriptions more likely to be provided if poor follow up anticipated (e.g. emergency room or walk-in clinic).	Training Restriction Environmental Restructuring Enablement

FMR = Family Medicine Resident

### Education and training (ET)

Education can address several barriers to appropriate prescribing. Residents identified the need to strengthen independent decision-making skills under the domain of goals. These goals can be addressed with education and training, including discussion of prognosis, complications associated with URTI, and management of patients with comorbidities or risk factors. Education may also help to improve confidence and understanding of consequences.

Residents identified the goal of improving their antibiotic knowledge. They also indicated that guidelines incorporating local resistance patterns would be particularly helpful. A set of up-to-date, evidence-based guidelines on the management of common primary care infections incorporating local resistance patterns might address this need. Education around these guidelines (i.e. rationale behind the recommendations, and clinical considerations when prescribing antibiotics) can help to meet residents’ learning goals.

Training residents on the use of delayed prescriptions may also help to decrease overall antibiotic consumption. The use of delayed prescriptions has been shown to decrease overall antibiotic use compared to immediate prescription ( Spurling *et al.*, 2017). This training will aid clinical decision-making and may address selected environmental barriers of overprescribing in the emergency room or walk-in clinics.

### Modelling shared decision-making with patients

The intervention function of modelling can address residents’ capabilities when managing patient preferences. Providing residents with the opportunity to observe shared decision-making and action plan development may increase confidence. Based on our study findings, additional scenarios to model include managing patients accustomed to receiving antibiotics from previous physicians; and addressing parent concerns about a sick child.

### Enablement - preceptor considerations

Attempts to change the prescribing practices of established physicians has had a modest impact, and a multi-faceted intervention may be required to elicit lasting improvement in prescribing ( Public Health England, 2015). Providing preceptors with an abbreviated version of the education being received by residents can facilitate teaching. Discussions between preceptors and residents should be encouraged when practice approaches differ. The opportunity for quality improvement projects in the office to reframe patient expectations of antibiotics may also be an opportunity to enable the resident.

### Resident Preferences for Educational Resources

When participants were probed about what educational resources would be useful to support their antibiotic prescribing practice, six preferred technology-based supports (e.g. mobile apps, websites), one preferred paper-based supports (e.g. posters, memos) and three chose a mix of both. The ideal product was identified as being reliable, capable of supporting clinical decision-making (e.g. diagnostic aids) and available at the point of care. Continuing medical education credits and preceptor discussions would also be helpful to supplement technical resources.

## Discussion

Studies that describe physician attitudes, beliefs and behaviours around antimicrobial prescribing have been called upon to inform the development of effective interventions to reduce overprescribing. Since antimicrobial stewardship principles are being taught widely in medical education ( Silverberg *et al.*, 2017), so too should these educational interventions be grounded in an understanding behaviours that underpin learners’ antibiotic prescribing. Using the TDF, this study found nine domains to be relevant to family medicine residents’ antibiotic prescribing practices: clinical decision-making; beliefs about capabilities; beliefs about consequences; social influences; professional role; environmental context; goals; and intentions.

Dallas *et al.* explored the attitudes of medical trainees in general practice and found that although learners recognized the importance of appropriate antibiotic prescribing, their practice of prescribing was at times incongruent with their knowledge base ( Dallas *et al.*, 2014). Our paper also found that residents wanted to ensure antibiotics were prescribed appropriately and to avoid harms associated with overprescribing. However, residents identified several barriers that limited their ability to meet this goal, including environmental considerations, beliefs about capabilities, and social influences. Papoutsi *et al.* found that clinical hierarchies influenced the context in which residents made antimicrobial prescribing decisions ( Papoutsi *et al.*, 2018). This context triggered residents’ responses (e.g. fear of criticism, need to appear competent) and affected subsequent prescribing decisions. Our paper also suggested that preceptors played an influential role on resident prescribing with preceptors modelling judicious prescribing to modelling prescribing that was more liberal or inconsistent with current recommendations. While these papers have explored residents’ attitudes, beliefs and values, our paper further contributes to this literature by classifying these factors into an established behaviour change framework which identified potential interventions for modifying behaviour.

In the area of antimicrobial stewardship, behavioural sciences have been incorporated into interventions to improve antibiotic prescribing among dentists ( Newlands *et al.*, 2016); to reduce antibiotic use in long term care ( Fleming *et al.*, 2014); and to encourage use of delayed prescribing for respiratory tract infections ( Sargent *et al.*, 2017). Our study contributes to the existing literature by using the TDF to describe factors affecting medical residents’ antibiotic prescribing behaviour for URTI and applying them to the BCW.

### Limitations

There were several limitations to this study. The residency program at North York General Hospital is a small program of approximately 30 residents and a small sample size was selected. However, thematic saturation was achieved. Comparisons between groups were not possible due to small sample size. Most residents were trained in Canada while there were a small number of IMG residents. This study also did not explore differences between residents in their first and second year of training.

## Conclusion

Using the TDF, this study identified several factors that influence antibiotic prescribing among family medicine residents, particularly in nine domains. Due to the complex nature of antibiotic prescribing for URTI, several intervention functions were identified as relevant to intervention design including modeling, enablement, education and persuasion. The selection of intervention functions to implement and the mode of delivery will depend on site-specific resources.

## Take Home Messages

• The attitudes, beliefs and values of family medicine residents around antibiotic prescribing are described using the Theoretical Domains Framework. Nine domains were identified as being relevant.
• The most prominent domains included social influence of the clinical preceptor and patient; goals; and beliefs about capabilities.
• Using the Behaviour Change Wheel, nine intervention functions were identified that could improve antibiotic prescribing behaviour among family medicine residents.
• The APEASE criteria was used to guide intervention selection. Education and training, modelling and enablement were three interventions that could address multiple domains and were feasible at our teaching site.

## Notes On Contributors

**Samantha Moe** is the Clinical Evidence Expert with the Knowledge Expert and Tools Program at The College of Family Physicians of Canada. She is an adjunct lecturer with the University of Toronto, with the Leslie Dan Faculty of Pharmacy and the Department of Family and Community Medicine. At the time of the study, Samantha was Clinical Pharmacist Practitioner with the Department of Family and Community Medicine at North York General Hospital. She is now working for the College of Family Physicians of Canada.

**Tiffany Kan** is the Antimicrobial Stewardship Pharmacist at North York General Hospital, Toronto, Ontario, Canada and an adjunct lecturer with the University of Toronto, Leslie Dan Faculty of Pharmacy.

**Charlene Soobiah** is a PhD candidate at the Institute for Health Policy, Management, and Evaluation at the University of Toronto. She specializes in health service research outcomes and evaluations.

**Azy Golian** is a practicing family physician in Toronto, Ontario, Canada. She completed her medical degree and residency training at the University of Toronto. She is also a faculty member with the University of Toronto and enjoys teaching medical students and residents.

**Timothy Li** is a community family physician practicing in Toronto, Ontario, Canada. He completed his medical undergraduate and residency training at the University of Toronto, and is appointed Lecturer with the University of Toronto, Department of Family and Community Medicine.

**Sumit Raybardhan** is the Infectious Disease Pharmacist at North York General and co-leads the institutional Antimicrobial Stewardship Program.
