# Supplementary material for: Using a behavioural framework to optimize antibiotic prescribing by family medicine residents
Source: MedEdPublish (2016). 2021 May 9;10:113. [Version 1] doi: 10.15694/mep.2021.000113.1 (PMC10939518; doi:10.15694/mep.2021.000113.1)
Supplement: Peer review history

# Reviewer report: Reviewer response for version 1

**DOI:** 10.21956/mep.18898.r26997

**Published:** 2021-08-03

**Authors:** Anita Samuel

This review has been migrated. The reviewer awarded 4 stars out of 5 This is a well-written article that addresses a clearly identified gap. The strength of this article is the methodology of the study. Appropriate frameworks and theories guided the study and using a qualitative research reporting tool is to be commended. With only 12 participants, the sample size is small but acceptable for qualitative studies. What is concerning is that the interviews only took an average of 24 minutes. This in combination with the small sample size raises questions about the claim of data saturation. You recruited 1st and 2nd years for this study. Was there a difference in the perceptions of these two groups? It might be interesting to explore this.

Reviewer Expertise:

NA

No decision status is available

# Reviewer report: Reviewer response for version 1

**DOI:** 10.21956/mep.18898.r26998

**Published:** 2021-05-09

**Authors:** Subha Ramani

**Affiliations:** 1 Harvard Medical School, Brigham and Women's Hospital

This review has been migrated. The reviewer awarded 4 stars out of 5 I read this article with interest as I agree with the authors about the importance of antibiotic stewardship. It is also correct to say that stewardship awareness should begin early for postgraduate clinical trainees. A behavioural lens to explore patterns of antibiotic use is quite appropriate and accordingly a qualitative study design is the right one. The authors have done an excellent job of mapping themes to domains of the TDF, the supplementary files include tables that clearly outline the findings along with representative quotes. I like the fact that the restuls include behaviours as well as potential interventions.Introduction- I am not entirely clear what the authors mean by modest effect- effect on what? I am also not sure “dozens of studies” adds anything, especially since 3 are cited. “Several studies” would suffice.Methods- This sentence sounds awkward- A qualitative study was performed using an iterative and thematic approach to code and analyze data. The introduction usually presents the approach- a qualitative study design using an inductive approach…Thematic analysis should instead be placed under data analysis.Why the authors choose to separately talk about IMGs is not clear to me, the authors could have explained why they chose to highlight this status.The rest of the methods section has been described clearly.I wonder if thematic saturation can be claimed on the strength of a small number of participants. This is also not required, authors could say they gathered sufficient data to inform their study questions.Data analysis should be a section under methods where details of how coding was done and how the thematic analysis, originally alluded to, was performed.Figure 1 is nicely done and informative.Results are very well writtenDiscussion- The authors list the small sample size as a limitation, rightly so. Yet, they state that thematic saturation had been achieved. I am not entirely sure this can be categorically stated in such a small number. However, it is not strictly necessary. The paper is well written, well referenced. The authors have used a creative framework to analyze antibiotic prescribing patterns and outlined potential interventions.All clinicians, who supervise and teach clinical trainees, would be interested in reading the paper. The paper could also provide ideas for other clinical teachers to explore to improve clinical skills of their postgraduate trainees.

Reviewer Expertise:

NA

No decision status is available
